# Current advances in the function and biogenesis of peroxisomes and their roles in health and disease

**DOI:** 10.1007/s00418-021-01982-1

**Published:** 2021-04-05

**Authors:** Noa Dahan, Tania Francisco, Christian Falter, Tony Rodrigues, Vishal Kalel, Markus Kunze, Tobias Hansen, Wolfgang Schliebs, Ralf Erdmann

**Affiliations:** 1 Department of Molecular Genetics, Weizmann Institute of Science, Rehovot, Israel; 2 Instituto de Investigação e Inovação em Saúde (i3S), Universidade do Porto, Porto, Portugal; 3 Plant Biochemistry and Infection Biology, Institute of Plant Science and Microbiology, University of Hamburg, Hamburg, Germany; 4 Department of Pathobiology of the Nervous System, Center for Brain Research, Medical University of Vienna, Spitalgasse 4, 1090 Vienna, Austria; 5 Department System Biochemistry, Institute of Biochemistry and Pathobiochemistry, Medical Faculty, Ruhr-University of Bochum, Universitätstr.150, 44780 Bochum, Germany

## Introduction

The 7th Open European Peroxisome Meeting (OEPM) was held on the 15th and 16th of October 2020 due to the Corona pandemic as ZOOM conference. The OEPM is a biannual meeting organized by European expert researchers in peroxisome biology. Previous meetings were held in Leuven, Belgium (2006), Lunteren, The Netherlands (2010), Dijon, France (2012), Neuss, Germany (2014), Vienna, Austria (2016), and Groningen, The Netherlands (2018). For the 2020 meeting nearly 200 participants registered from 10 European countries, as well as Israel, Canada, the USA, Japan, Russia, and South Korea.

At the OEPM, it is tradition that only young peroxisome researchers (PhD students or junior Post-docs) present their work to an international audience. At this year’s meeting, 46 talks were presented in eight sessions. During the meeting, the OEPM 2020 Peroxisome Research Young Investigator Award (sponsored by Ruhr-University of Bochum) was awarded. This award is given to the early career researcher, who was first author of the best publication in the peroxisome field over the last 2 years. At this meeting, the award was given to **Victoria Riccio** from the group of Peter Kim at the University of Toronto (Canada). The awarded paper describes that the deubiquitinating enzyme USP30 prevents pexophagy by counteracting the action of the peroxisomal E3 ubiquitin ligase PEX2. They also show that USP30 can rescue the peroxisome loss observed in some disease-causing peroxisome mutations, pointing to a potential therapeutic target (Riccio et al. 2019). Victoria Riccio received the prize during the meeting and presented the Young Investigator Award lecture about the paper. Members of the jury were Myriam Baes (Leuven), Mustapha Cherkaoui-Malki (Dijon), Ralf Erdmann (Bochum), Marc Fransen (Leuven), Aurora Pujol (Barcelona), Sigrun Reumann (Hamburg), Maya Schuldiner (Rehovot), Ron Wanders (Amsterdam), Bettina Warscheid (Freiburg), Hans Waterham (Amsterdam), and Einat Zalckvar (Rehovot).

**Iulia Revenco** (KU Leuven, Belgium) and **Marc Pilegaard Pedersen** (University of Groningen, The Netherlands) presented the Marie Skłodowska-Curie Innovative European Training Network ‘PerICo’ (Peroxisome Interactions and Communication) that is coordinated by Prof. Dr. Ida van der Klei (University of Groningen). PerICo brings together ten full partners and five partner organizations, from seven European countries, together with Israel and Canada, and fosters the education of 15 PhD-students in projects aimed at uncovering how peroxisomes participate in cellular interactions and signaling.

In the evening session, **Andrew Longenecker** presented ‘Diego’s story’, the case of his son Diego, who suffers from a PEX10 Zellweger Spectrum Disorder (ZSD), a peroxisome biogenesis disorder (PBD). In his presentation, he reported on a non-profit PBD-project (DiegoZSDResearch@gmail.com) that he and his wife created to engage with the research community and other parents.

Peroxisomes are ubiquitous cell organelles of eukaryotic cells. They are surrounded by a single membrane and harbor a highly variable enzyme composition that determines the function of these multi-purpose organelles. Common functions of peroxisomes include β-oxidation of fatty acids and containment of pathways that produce hydrogen peroxide, which is then detoxified by catalase. Examples for species-specific peroxisomal pathways are alpha-oxidation of branched-chain fatty acids and synthesis of plasmalogens in humans, and contribution to the synthesis of penicillin in fungi or bile acids in humans (Wanders and Waterham 2006). Defects in peroxisomal enzymes or biogenesis of the organelle result in peroxisomal disorders, like X-linked Adrenoleukodystrophy or the Zellweger syndrome, respectively (Waterham et al. 2016). There is an increasing interest in peroxisome research, which is fueled by the fact that major questions concerning the biology of these fascinating organelles are still unanswered. Such as the import of folded, even oligomeric proteins into peroxisomes with the underlying mechanism still being unresolved (Baker et al. 2016; Walter and Erdmann 2019). Our knowledge on the biogenesis, dynamics (fission, transport), degradation by autophagy (pexophagy), etc. is still scarce, leaving room for exciting discoveries (Costello and Schrader 2018; Islinger et al. 2018; Mast et al. 2020; Mahalingam et al. 2020). Moreover, peroxisomes function in concert with other organelles, including metabolic interaction and signal transduction. In this context, an emerging theme is the investigation of physical contacts of peroxisomes and mitochondria, lipid droplets, vacuoles (lysosomes), and the ER via contact sites (Shai et al. 2016; Islinger et al. 2018; Farré et al. 2019; Schrader et al. 2020). Moreover, glycosomes, as peroxisomes of trypanosomatid parasites are called (due to the localization of glycolytic enzymes) have been identified as suitable targets for novel leads compounds for therapy of diseases like African sleeping sickness, Chagas Disease and Leishmaniasis (Dawidowski et al. 2017). Another reason for the steadily increasing interest is the perception that the cellular duties of these fascinating organelles go well beyond their undoubted metabolic functions. Peroxisomes turned out to have important non-metabolic roles, such as their function as signaling platform in antiviral response and thus their contribution to innate immunity (Ferreira et al. 2019), and their contribution to cooperative cell defense mechanisms against oxidative stress (Fujiki et al. 2020). Moreover, peroxisomes are critical contributors to ageing, longevity (Deori et al. 2018) and age-related disorders, including Alzheimer’s disease and cancer (Berger et al. 2016; Islinger et al. 2018; Dahabieh et al. 2018).

Below is an overview of the sessions and talks of this very successful and inspiring OEPM. The order of talks at the meeting were essentially ordered alphabetically.

## Session 1: Chair: Noa Dahan, Weizmann Institute of Science, Israel

Due to the ZOOM format of this year’s OEPM the first session was somewhat shuffled, the speakers took us on a peroxisome tour, in which we learned about different aspects of peroxisome biology from structural studies to protein targeting and peroxisomal diseases studied in a variety of model systems.

The first talk was given by **Pascal Lill** (Christos Gatsogiannis Laboratory, Max Planck Institute of Molecular Physiology, Dortmund, Germany), who presented a structural characterization of the main components of the peroxisomal docking complex in *S. cerevisiae*, Pex14 and Pex17. Pascal and his colleagues applied cryo-electron microscopy single particle analysis and cryo-electron tomography on purified complexes and found that three copies of Pex14p and a single copy of Pex17p assemble to form a 20 nm rod-like particle (Lill et al. 2020). The different subunits are arranged in a parallel manner, showing interactions along their complete sequences and providing receptor-binding sites on both membrane sides. The long rod facing the cytosol is mainly formed by the predicted coiled-coil domains of Pex14p and Pex17p, possibly providing the necessary structural support for the formation of the import pore. These high-resolution images enabled a glimpse into the architecture of the complex and Pascal discussed the possible implications of these observations on our understanding of proteins translocation from the cytosol into the lumen of the peroxisome.

We than moved on to learn about targeting of one of the major inhabitants of peroxisomes, the antioxidant enzyme catalase. In Arabidopsis, there are three isoforms of this enzyme from which CAT2 is the most important for photorespiration. **Yousef Al-Rhoom Al-Hajaya** (Alison Baker laboratory, University of Leeds, UK) discussed his findings regarding the import of Catalase 2 into peroxisomes of Arabidopsis. To determine the importance of the last 18 amino acids of CAT2 to the import into peroxisomes, Yousef has created variants with modified C-termini that were introduced into cat2-1 mutant cells. Using morphological and biochemical assays, Yousef showed that the wild type phenotype was largely restored in all of the lines transformed with CAT2 variants. Based on his observations, Yousef concluded that despite a high degree of sequence conservation of the C-terminal region of CAT2, the last 18 amino acids of this protein are not important for catalase activity.

Peroxisomes are involved in multiple metabolic and catabolic cellular processes. Mutations in different peroxisomal genes have various effects on peroxisomal function; therefore, peroxisomal disorders have a broad spectrum of clinical forms that differ in their severity. When it comes to studying peroxisomal disorders, the biggest challenge is to associate a specific peroxisomal function with a clinical symptom. The next three talks aimed to assign a physiological phenotype with specific peroxisomal gene deletions by using different model organisms.

**Sai Kocherlakota** (Myriam Baes laboratory, KU Leuven, Belgium) presented his research focusing on the destructive effect of β-oxidation deficiency on the shape and function of the retinal pigment epithelium (RPE) cells, causing visual abnormalities (Das and Baes 2019). Sai used a mouse model with RPE-specific deletion of MFP2, a central enzyme in peroxisomal beta-oxidation, and found that they exhibit a loss of RPE hexagonal shape and RPE protrusion into the photoreceptors, but no general shortening of POS. The mice also showed increased retinal distress, inflammation and reduced retinal function. He further proved that the RPE of global MFP2 knockout mice undergo dedifferentiation by showing a reduction in RPE65 and increased cell proliferation. He finally concluded that peroxisomal beta-oxidation in the RPE is crucial to maintain the integrity of this cell layer, and secondarily of the neural retina.

**Sushil Bhandari** (Seong‐Kyu Choe laboratory, Wonkwang University School of Medicine, South Korea) presented the outcome of deleting PEX5, an essential peroxisomal protein targeting receptor, in zebrafish. Sushil and colleagues observed a series of phenotypes that resembles those of human patients that suffer from Zellweger syndrome. The mutated zebrafish were highly sensitive to starvation, which led to early larval mortality due to mitochondrial abnormalities, TORC1 activation and autophagy repression. Sushil has also indicated that the drugs that ameliorate such conditions partially rescues the mutant phenotype, which may provide therapeutic options for treating peroxisomal diseases in human patients.

The last talk was given by **Margret H. Bülow** (The Life and Medical Sciences Institute of the University of Bonn, Germany) who presented the results of a research conducted in her laboratory that studies nutrient signaling of organelles in *Drosophila melanogaster*. Drosophila mutants for the peroxisome assembly factors, peroxins, phenocopy the symptoms of patients suffering from Peroxisomal Biogenesis Disorders. Margret Bülow talked about their recent work on protein catabolism in a Peroxin (Pex) 19 mutant. Pex19 mutants show dysregulated ceramide synthase activity, which leads to lipid catabolism, accumulation of free fatty acids and mitochondrial hyperactivity (Sellin et al. 2018). Analysis of the Pex19 mutant lipidome revealed that due to the accumulation of very long-chain fatty acids that is characteristic for peroxisome dysfunction, ceramide-derived sphingolipids incorporate longer fatty acids. This alters the nutrient signaling pathways of Pex19 mutants: while insulin/TOR signaling is repressed, lipid and protein catabolism are upregulated, leading to depletion of lipid stores and muscle atrophy. This suggests that the altered fatty acid profile of Pex19 mutants affects their perception of the nutritional status of the organism.

## Session 2: Chair: Tania Francisco, Universidade do Porto, Portugal

Peroxisomes, initially named microbodies, were aptly renamed by De Duve and colleagues for their content in enzymes involved in the production and degradation of hydrogen peroxide. Since then, many other metabolic pathways and functions have been ascribed to peroxisomes, and strikingly, more than 50 years later from their discovery, this list is still increasing. One example, was provided by the Peroxisome Research Award lecture winner, **Eden Yifrach** (Schuldiner lab, Weizmann Institute of Science, Department of Molecular Genetics, Rehovot, Israel), who showed us her findings on new peroxisomal functions. Eden presented recent and exciting new data obtained from a high content screen on a collection of ~ 6000 yeast *Saccharomyces cerevisiae* strains, which identified ~ 40 potential new peroxisomal proteins. As an example, Eden showed that two subunits of an ubiquitin ligase involved in the proteasomal degradation of gluconeogenic enzymes are targeted to peroxisomes by Pex5 in gluconeogenic conditions, which results in the accumulation of gluconeogenic enzymes in the cytosol. These findings add a new role to peroxisomes in the regulation of gluconeogenesis. The identification and validation of the other new potential peroxisomal proteins promises new discoveries on other peroxisomal functions.

To fulfill such a variety of functions, a dynamic flow of metabolites in and out of peroxisomes must exist. As such, transporters, channels or pores should, in principle, facilitate these transfers. Two talks of this session addressed this issue. Considering that peroxisomes entail several ATP-dependent processes, and that no ATP-generating enzymes have ever been identified inside peroxisomes, **Serhii Chornyi** (Waterham lab, Laboratory Genetic Metabolic Diseases, Academic Medical Center, Amsterdam, The Netherlands) showed us recent results focused on how ATP reaches the peroxisomal lumen. The results obtained using peroxisome-targeted fluorescent ATP indicator proteins and CRISPR generated cell models with selective deletion of single or several genes, suggested that ABCD (ATP-binding cassette subfamily D) transporters may function as ATP transporters. However, after deleting ABCD1, SLC25A17, PXMP2, or PEX11B genes, the kinetics of ATP transport across the peroxisomal membrane were not affected, suggesting that functional redundancy may be an important property of peroxisomal metabolite transport. Also focusing on how metabolites cross the peroxisomal membrane, **Cláudio Costa** (Fransen lab, Laboratory of Lipid Biochemistry and Protein Interactions, KU Leuven, Belgium) updated us on his recent findings on the potential role of human Peroxisomal Membrane Protein 34 (PMP34), a peroxisomal membrane protein belonging to the mitochondrial solute carrier family whose function is still unclear. Using a generated PMP34-deficient cell line and ratiometric fluorescent sensors, redox parameters such as the H_2_O_2_ levels, GSSG/GSH and free NAD^+^ /NADH ratios were measured in the cytosol, mitochondria, and peroxisomes, and a role for PMP34 in the peroxisomal redox metabolism was evaluated. Although the gathered data excluded a role in the channeling of H_2_O_2_, they revealed that PMP34 is indeed involved in peroxisomal redox regulation by a yet unknown mechanism, requiring further investigations.

In the last talk of this session, **Sandipan Dasgupta** (Schuldiner lab, Department of Molecular Genetics, Weizmann Institute of Science, Israel) shared exciting results on the regulation of peroxisome biogenesis. Using a 2D co-culture model of patient-derived cells harboring a mutant form of PEX6 and wild-type immortalized cells, he demonstrated that the peroxisome-deficient phenotype could be complemented. Furthermore, complementation was not mediated by peroxisome transfer between cells, but more likely by the transfer of PEX6 mRNA from donor to mutant acceptor cells and translation therein, suggesting that mRNA-mediated horizontal gene transfer may be responsible for the complementation of the mutant phenotype. As such, mRNA-mediated horizontal gene transfer mechanism may constitute a potential mechanism for organelle biogenesis regulation through intercellular communication.

## Session 3: Chair: Christian Falter, Plant Biochemistry and Infection Biology, University of Hamburg, Germany

In the 3rd session on novel peroxisome research from different fields, six highly interesting talks were reported, five of which are summarized here. Ranging from yeast via plants to animals, the function, structure and membrane topology of important proteins involved in transport processes across the peroxisomal membrane and between organelles and the role of peroxisomes in cancer and blood vessel development was discussed.

Proteins of the ATP-binding cassette (ABC) transporter subfamily D are integral membrane proteins that transport diverse substrates into peroxisomes. In the model plant *Arabidopsis thaliana*, Comatose (CTS) is responsible for the delivery of substrates for peroxisomal fatty acid β-oxidation. During the transport process, fatty acyl-CoA substrates are cleaved by the transporter’s intrinsic thioesterase activity. Because CTS does not possess an annotated thioesterase domain, the cleavage of fatty acyl-CoA substrates by CTS remains mysterious. **Jack Wright** (Alison Baker laboratory, University of Leeds, UK) presented his progress in determining the structure of CTS by cryo-electron microscopy. He was able to express and purify wild-type CTS and an inactive mutant version, and subjected both proteins to preliminary cryo-EM studies. His work provides the basis for future experiments aiming at solving the structure of CTS at high resolution.

The data presented in the second talk by **Tanja Eberhart** (Werner Kovacs laboratory, ETH Zurich, Switzerland) address the role of peroxisomes in the most common type of kidney cancer, the so-called clear cell renal cell carcinoma. In the corresponding ccRCC cell lines, a peroxisomal membrane protein fused at the cytosolic side with ubiquitin was used to trigger pexophagy. Tumor growth and size were significantly reduced after peroxisome depletion in a xenograft mouse model, which is in line with a previous finding that high-grade human ccRCCs contain a higher number of peroxisomes. Further studies revealed an increased collagen deposition in histological samples of tumors derived from the same cell lines, and multi-omics analyses uncovered changes in specific metabolic pathways that are important for cell proliferation and tumor progression. The data hint towards an accumulation of biologically active lipids or toxic intermediates in the ubiquitin-tagged ccRCC cell lines due to peroxisome deficiency that leads to fibrosis and an impairment of tumor metabolism. Taken together, this study provides new insights into peroxisome functions in cancer progression and offers the basis to study novel treatment strategies for kidney cancer.

To elucidate the import mechanism of matrix proteins, the importomer and its interplay with the cytosolic receptors is in focus. Two main components of the docking complex, PEX13 and PEX14, are suggested to form, together with PEX5, the import channel. **Maria Joao Ferreira** (Jorge Azevedo laboratory, University of Porto, Portugal) presented data to better define the membrane topologies of the two peroxins and better understand how this protein complex is organized. PEX14 comprises a small N-terminal domain, a transmembrane α-helix, and a C-terminal coiled-coil domain, while PEX13 contains three putative transmembrane domains followed by a Src homology 3 domain. The topologies of these two membrane proteins were determined by subjecting proteoliposomes and rat liver peroxisomes to protease protection assays and analyzing the protease-protected fragments by mass spectrometry, Edman degradation and Western blotting. The data revealed that the N-terminus of PEX14 is exposed to the peroxisomal matrix and the C-terminal coiled-coil domain to the cytosol. Unexpectedly, the N- and C-termini of PEX13 were located on opposite sides of the peroxisomal membrane as compared to PEX14, with the C-terminal Src homology 3 domain facing the organelle lumen (Barros-Barbosa et al. 2019).

**Johannes Freitag** (Michael Bölker laboratory, Philipps-University Marburg, Germany) reported his latest findings about the dual distribution of the phosphatase Ptc5 between mitochondria and peroxisomes in *Saccharomyces cerevisiae*. The integral membrane protein has an N-terminal mitochondrial and C-terminal peroxisomal targeting signal (PTS1). Ptc5 is first targeted to the inner membrane of mitochondria by its transmembrane domain and only subsequently redirected to peroxisomes. In mitochondria, Ptc5 is cleaved downstream of the TMD by the mitochondrial inner membrane peptidase complex, releasing the soluble Ptc5 domain into the intermembrane space or into the peroxisomal matrix (Stehlik et al. 2020). In a microscopy screen, Johannes identified components of the ERMES complex (Endoplasmic Reticulum and Mitochondria Encounter Structures) as regulatory factors required for dual targeting of Ptc5. Deletion of ERMES components reduced initial mitochondrial import of Ptc5 and consequently also its sorting to peroxisomes. Targeting of other dually localized proteins was also affected in the same ERMES mutants. In addition, it was observed that overexpression of several dually targeted proteins enhanced the physical association between mitochondria and peroxisomes. This association depended on the presence of ERMES proteins. Johannes suggested a novel model for peroxisome–mitochondria contact site formation based on the import of dually targeted proteins. Mitochondrial and peroxisomal targeting signals in dually targeted proteins are proposed to compete with each other by simultaneously employing components of both, the peroxisomal and mitochondrial import system.

**Melissa García-Caballero** (Peter Carmeliet laboratory, KU Leuven, Belgium) discussed the functions of peroxisomes in blood endothelial cells (ECs). Their cellular metabolism controls angiogenic processes, i.e., the development of new blood vessels from preexisting ones, but the contribution of peroxisomal metabolism to this process is yet unknown. In PEX5 silenced human ECs and mice with PEX5 deficiency specific for ECs, differences in very long-chain fatty acid and plasmalogen levels and in phospholipid composition were detected. In both experimental models, angiogenesis was reduced. Additionally, other pathways like mitochondrial fatty acid β-oxidation, TCA cycle, oxidative phosphorylation and nucleotide synthesis were altered. Thereby, Melissa provided novel insights into the importance of peroxisomes in EC growth and maintenance.

## Session 4: Chair: Tony Rodrigues, Universidade do Porto, Portugal

In the fourth session, PEX proteins, i.e. peroxins, retake center stage. These are the main effector proteins controlling all aspects of peroxisome biogenesis and errors within their sequence can have devastating consequences in peroxisomal function and health (Waterham and Ebberink 2012). To date 37 peroxins have been identified among all eukaryotic organisms and, although about half of these peroxins are absent in higher eukaryotes, many of these more divergent PEX orthologs still share some degree of conservation and/or functional relation to their human counterparts (Dodt et al. 2001; Schliebs and Kunau 2006).

On that note, **Renate Jansen** (Ida van der Klei laboratory, University of Groningen, The Netherlands) presented a comparative genomics study of PEX genes. In addition to identifying previously unknown PEX orthologs, other interesting observations were also shared. For example, it was shown that a typical feature of PEX19 (the CAAX box) present in all Fungi, Plantae and Animalia, is absent in most Protists. Differences could also be found among the same phyla, with *Saccharomyces cerevisiae* possessing several PEX genes that are otherwise absent from other fungi, while also lacking other fungi-specific PEX orthologs. The work presented was also meant to rekindle a larger conversation about the standing nomenclature of these PEX proteins, which is often not consistent with their evolutionary relationship. In an ensuing breakout room discussion, Ida van der Klei presented us with some ideas of how naming could be changed to better reflect conservation or even function.

Speaking of function, most peroxins are involved in the import of peroxisomal proteins into the organelle matrix (reviewed in (Francisco et al. 2017). Central players of this import machinery include the cytosolic receptor PEX5, and the multi-peroxin complex that forms the docking/translocation module at the peroxisome membrane. PEX13 and PEX14, two peroxins that strongly interact with each other and PEX5, are integral components of this module (Otera et al. 2002; Fransen et al. 2002). To this day, the structure of this protein channel has not been elucidated.

**Stefan Gaussmann (**Michael Sattler laboratory, Munich, Germany) shared with us some structural data on PEX13 and its interaction with PEX5 and PEX14. He described a novel motif of PEX13 that interacts with PEX14. Additionally, he showed that this new motif might negatively regulate the interaction of the SH3 domain with some of the pentapeptide motifs of PEX5. The data point to the existence of an intricate interplay of protein–protein interactions between PEX5, PEX13, and PEX14 regulating protein import into the peroxisomal matrix.

As alluded to above, mutations in genes encoding peroxins are at the root of the so-called peroxisomal biogenesis disorders. The most prominent, the Zellweger spectrum disorders (ZSD), can emerge from mutations in 13 PEX genes, but mutations in PEX1 are by far the most prevalent (Waterham and Ebberink 2012).

Lingxiao Chen and Catherine Argyriou (Nancy Braverman laboratory, McGill University, Montreal, Canada) presented two studies using a mouse model homozygous for the PEX1-G844D allele, the murine equivalent of a very common mutation causing ZSD. This mouse model recapitulates many of the hepatic features and retinopathy present in these patients (Hiebler et al. 2014). First, presented by **Lingxiao Chen**, came an assessment of the efficacy of colic acid therapy in preventing/treating liver disease caused, in part, by bile acid synthesis defects that result from a disrupted peroxisomal matrix protein import (Berendse et al. 2019). They found that diet supplementation with cholic acid reduced levels of toxic C27-bile acid precursors and led to elevated levels of mature C24-bile acids in a dose dependent manner. In fact, mitochondrial oxidative stress was reduced at all doses, while necrosis of hepatocytes was alleviated by 0.1% cholic acid in older cohorts. However, both intermediate and high doses of cholic acid (0.1, 0.2 and 0.5%) further enhanced growth restriction and liver dysfunction, suggesting hepatotoxicity due to overdose. Peroxisome number and peroxisomal import could not be improved at any dosage, but small doses of cholic acid might still retain some therapeutic potential.

Last, but not least, **Catherine Argyriou** presented data on a proof-of concept trial for PEX1 retinal gene therapy using the aforementioned mouse PEX1-G844D model. Following vector delivery, retinas expressed human PEX1 without exhibiting gross histologic side effects. 8–20 weeks post injection, retinal function and visual acuity was improved. After 6–7 months, electroretinogram response continued to show improvements relative to controls, and the ZSD archetypal C26-fatty acid accumulation was markedly reduced in whole retinal lysates. Additionally, the authors developed a MALDI-imaging MS technique to resolve the distribution of peroxisome-mediated lipids along retinal sections. This will permit assessing metabolic recovery at distinct tissue regions following gene delivery without contamination by non-transduced areas.

## Session 5: Chair: Vishal Kalel, Ruhr-University Bochum, Germany

Eukaryotic cells compartmentalize various functions in different cellular organelles, but for metabolite transfer and efficient inter-organelle communication, they need to come into close physical contact of each other (“hugging”). **Suzan Kors** (Michael Schrader laboratory, University of Exeter, UK) presented their work on regulation of peroxisome–ER interactions in mammalian cells. Recently, they identified that peroxisomal acyl-CoA-binding domain proteins ACBD4 and ACBD5 interact with ER-resident VAMP-associated proteins (VAP) which mediate peroxisome–ER membrane contact sites (MCS) (Costello et al. 2017a, b). The VAP family proteins are involved in tethering the ER to other organelles via interaction with FFAT [two phenylalanines (FF) in an Acidic Tract] (-like) motif-containing proteins such as peroxisomal ACBD4/5. To study how peroxisome-ER contacts are regulated, they identified various phosphorylation sites in ACBD4 and ACBD5, which differently regulate the interaction with VAPB, and hence, modulate peroxisome–ER interactions. They are further investigating how kinases/phosphatases act on the ACBD5-VAPB tether and studying different conditions that affect peroxisome–ER contacts.

To study the molecular mechanism underlying pexophagy, **Hongli Li** (Marc Fransen laboratory, KU Leuven, Belgium) reported development and validation of a robust and sensitive cell line (DD-myc-DAO Flp-In T-REx HEK-293 background) stably expressing a peroxisome-targeted variant of mKeima (po-mKeima). mKeima is a pH-sensitive red fluorescent protein that exhibits a dual excitation (peak at neutral pH: 440 nm; peak at acidic pH: 586 nm) but single emission (peak: 620 nm) spectra. These ratiometric pH sensing properties can be used to monitor the lysosome-based degradation of subcellular structures in living cells. By employing this reporter cell line, they confirmed that pexophagy can be triggered by heterologous expression of PMP34-Ub, PEX5-HaloTag, or PEX3(1–44)-EGFP. The study also corroborated that amino acid starvation and recultivation in nutrient-rich medium promotes peroxisome degradation. However, externally added or peroxisome-derived H_2_O_2_ did not induce pexophagy, which disagrees with a previous report (Zhang et al. 2015). This robust and sensitive cell model serves as new tool to identify and study pexophagy-linked factors at the cellular and molecular level.

Trypanosomatid parasites are responsible for devastating African sleeping sickness, Chagas disease, and Leishmaniasis that cause around 18 million human infections globally. **Mengqiao Li** (Ralf Erdmann laboratory, Ruhr-University Bochum, Germany) reported the identification of the highly divergent trypanosomal PEX3, which is the central component of the machinery of glycosomal membrane import and the master regulator of peroxisome biogenesis (Kalel et al. 2019). Due to the very low degree of sequence conservation, Trypanosoma PEX3 was identified with a combinatory approach of affinity isolation and Mass spectrometry analysis of PEX19-interacting proteins, and secondary structure homology screening. Mengqiao showed that Trypanosoma PEX3 directly interacts with PEX19, localizes to glycosomes in vivo, and that knock-down of PEX3 expression by RNA interference causes mislocalization of glycosomal matrix and membrane proteins leading to a severe growth defect. Differences in the parasites and human PEX3-PEX19 interaction interface and essentiality of PEX3 for parasite survival indicated that Trypanosoma PEX3 could be a novel drug target against trypanosomiasis. In this direction, she is pursuing establishment and optimization of an in vitro screen.

Although detoxification of hydrogen peroxide (H_2_O_2_) is the hallmark function of peroxisomes, H_2_O_2_ also serves as a signaling messenger. To elucidate the poorly understood regulatory role of peroxisomes in H_2_O_2_ signaling events, **Celien Lismont** (Marc Fransen laboratory, KU Leuven, Belgium) described a proteomics-based approach to catalog protein thiol targets of hydrogen peroxide derived from peroxisomes. They combined their previously developed human cell system in which peroxisomal H_2_O_2_ production can be controlled (Lismont et al. 2019) with a yeast AP-1-like (YAP1)-based redox proteomics strategy established by the Van Breusegem group in *Arabidopsis thaliana* (Waszczak et al. 2014). They optimized and modified the YAP1-based probe for sulfenome mining in the cytosol, mitochondria, or peroxisomes. Using this unbiased approach, they were able to identify specific and common targets of peroxisome-derived H_2_O_2_ and externally added H_2_O_2_. Celien also showed that they can distinguish between the targets in different cellular locations and, in many cases, they could identify the oxidatively modified cysteines. This strategy enables gathering important information about the primary protein thiol targets of peroxisome derived H_2_O_2_, thereby gaining a better understanding of the role of peroxisomes in cellular redox signaling networks.

The talk by **Renate Maier** (Bettina Warscheid laboratory, University of Freiburg, Germany) addressed the question if phosphorylation of Pex14p plays a role in regulating peroxisomal matrix protein import. They established a comprehensive Pex14p in vivo phosphorylation map of *Saccharomyces cerevisiae* using high-resolution mass spectrometry (Schummer et al. 2020). Their analysis of single phosphomimicking and non-phosphorylatable Pex14p variants revealed that a phosphomimetic mutation of Pex14p at serine 266 results in impaired growth and reduced peroxisomal import of citrate synthase 2 (Cit2p). Cit2p is an enzyme of the glyoxylate cycle, which is required to build carbohydrates from acetyl-CoA when cells are grown under non-fermentable conditions. She showed that phosphomimetic and non-phosphorylatable Pex14p-S266 mutants show reverse growth phenotypes in oleic acid and ethanol, when acetyl-CoA is produced (and Cit2p activity is required) in peroxisomes and the cytosol, respectively. Consistent with their data for the Pex14p phosphosite mutants, in vivo phosphorylation at S266 was increased upon growth in ethanol as compared to oleic acid. Taken together, their data show that phosphorylation of Pex14p-S266 is a switch mechanism to adjust the subcellular distribution of Cit2p to the metabolic requirements of the cell.

## Session 6: Chair: Markus Kunze, Center for Brain Research, Medical University of Vienna, Austria

The talks of the 6th session dealt with regulatory processes acting on the transport of a cofactor (NAD^+^), an inducible interaction between a receptor protein and a novel cargo protein or the transport of entire peroxisomes along the cytoskeleton, but also with the structural dynamics in the receptor PEX5 or an unexpected inclusion of a peroxisomal protein into a cytosolic ribosomal protein complex.

**Sophie Moul** (Stuart Warriner laboratory, University of Leeds, UK) introduced a novel and innovative approach to artificially redirect proteins from the cytosol into peroxisomes by initiating a piggy-back like approach. Adding a strong type-1 peroxisomal targeting signal (PTS1) to a domain or a chemical compound with high affinity to another protein (PrA) should generate a linker interconnecting PrA with the PTS1-receptor PEX5 and thus initiating its import into peroxisomes. As peroxisomes are constantly subjected to turnover by pexophagy, PrA will finally be destroyed. Using the halotag-domain extended by streptavidin as reporter protein, Sophie demonstrated that a halotag ligand equipped with a PTS1 was able to mediate its interaction with PEX5 and other peroxins. Furthermore, the first steps to demonstrate the functional capability of this approach in living mammalian cells were introduced. This type of conditional transport into peroxisome mimics the well-established PROTACs (Proteolysis Targeting Chimeras) system mediating cytosolic protein degradation by the proteasome, but acts in a completely independent manner via lysosomal degradation.

Next, **Thomas Walter** (Ralf Erdmann laboratory, Ruhr University Bochum, Germany) described the surprising finding that thiolase from the yeast (*Sc*Fox3p) showed an unexpected sedimentation behavior comparable to a high-molecular complex in a density gradient, independently whether studying residual non-imported cytosolic proteins or the entirety of Fox3p in cells with dysfunctional peroxisomal import. This behavior was independent of the receptor (Pex7p) and co-receptor (Pex18p/Pex21p) proteins and could be recapitulated using bacterially expressed and isolated Fox3p, but was affected by the Mg^2+^ level and RNAse treatment. Furthermore, components of ribosomes were found among the proteins co-isolated with Fox3p and co-migrated in exclusion chromatography suggesting that Fox3p is part of a ribosome-associated complex of yet unknown function.

**Anastasija Plett** (Nicole Linka laboratory, Heinrich-Heine University Düsseldorf, Germany) investigated the peroxisomal membrane protein PXN from *A. thaliana*, which mediates the import of NAD^+^ in exchange for AMP. Specifically, she studied the relevance of a phosphorylated serine identified by phospho-proteomics by introducing either an inactivating point mutation (S → A) or a phospho-mimetic one (S → D) into the transporter protein. For this purpose, Anastasija took advantage of a deletion strain of the yeast *S. cerevisiae*, in which the ability to effectively degrade fatty acids depends on the import of NAD^+^ from the cytosol, because deletion of peroxisomal malate dehydrogenase (*Δmdh3*) ablates intraperoxisomal reoxidation of NADH. Expressing native plant PXN each of the mutated variants in this yeast strain revealed that PXN harboring the S → D mutation has higher transporter activity and this suggests that NAD^+^ transport is regulated in *A. thaliana*.

The focus of the work of **Julia Ott** (Ralf Erdmann laboratory, Ruhr University Bochum, Germany) was the protein ATAD1 (ATPase family AAA domain-containing protein 1), which is found at peroxisomes and mitochondria. ATAD1 contributes to the quality control of mitochondrial membrane proteins by extracting mislocalized non-mitochondrial proteins, but also by clearing stalled precursor proteins from the mitochondrial TOM translocase. When searching for interaction partners of ATAD1, various proteins of the peroxisomal import machinery were identified including PEX5, but also PEX1 and PEX6 involved in the recycling of PEX5 from peroxisomes into the cytosol. Using a human cell line, in which either ATAD1 or PEX1 or both genes were inactivated by CRISPR, Julia found that the lack of ATAD1 stabilizes PEX5 in PEX1-deficient cells. This suggests that ATAD1 is involved in the removal of PEX5 from the peroxisomal membrane, when its normal extraction by PEX1 is dysfunctional.

**Katharina Reglinski** (Christian Eggeling laboratory, Friedrich-Schiller-Universität Jena, Germany) described a surprisingly low diffusion rate of the large isoform of PEX5 extended by a SNAP-tag, when performing fluorescence correlation spectroscopy (FCS) in mammalian cells, which normally indicates an excessively large size of the PTS1-receptor. Excluding the contribution of cargo binding, of an interaction with membranous structures or of receptor oligomerization, Katharina retraced the large apparent size to the unstructured properties of the N-terminal half of PEX5. Surprisingly, this property was not found, when performing FCS-analysis of the corresponding PEX5 protein from protozoa (*Trypanosoma brucei*), which suggests either the binding of other human proteins specifically to the human N-terminal part of PEX5 or an atypical property in the N-terminal part of human PEX5, which is not evolutionary conserved.

Finally, **Maren Reuter** (Ralf Erdmann laboratory, Ruhr University Bochum, Germany) described her study on an N-terminal domain (NTD) of human PEX14, which interacts with PEX5 as well as with β-tubulin, thus placing PEX14 at the cross-road between protein import and organellar movement. Using a biochemical in vitro assay, the direct competition of PEX5 and microtubuli for the PEX14-NTD was demonstrated, which is regulated by phosphorylation. As phosphoproteomics identified the phosphorylation of a specific serine (S44) within the NTD of PEX14, Maren generated a non-phosphorylatable variant (S → A) and a phosphomimetic one (S → D). Only the latter caused a marked reduction in the affinity to microtubuli and thus to the cytoskeleton, without affecting the binding to PEX5. This suggests that in living cells a regulatory process can specifically modulate the attachment of peroxisomes to the cytoskeleton without affecting protein import.

## Session 7: Chair: Tobias Hansen, Ruhr-University Bochum, Germany

**Iulia Revenco** (Marc Fransen laboratory, KU Leuven, Belgium) presented her recent findings about the role of peroxisomes as hydrogen peroxide signaling hubs. A unique Flp-In T-REx 293 cell model was presented in which the production of peroxisomal, mitochondrial or cytosolic H_2_O_2_ is controlled in a time- and dose-dependent manner (Lismont et al. 2019). Electrophoresis mobility shift assays revealed a list of selected proteins susceptible for the oxidation of peroxisome-derived H_2_O_2_. Thereby, Iulia demonstrated that the extent of oxidatively modified proteins by peroxisome derived H_2_O_2_ is strongly influenced by multiple components of cell culture medium, like serum and glucose. Thus, changes in peroxisomal H_2_O_2_ metabolism might affect specific signaling pathways.

New investigations on the selectivity and the pathway of Pex9p-mediated matrix protein import were presented by **Markus Rudowitz** (Ralf Erdmann laboratory, Ruhr-University Bochum, Germany). Markus characterized the import of the highly oleate-inducible alternative PTS1-receptor Pex9p (Effelsberg et al. 2016; Yifrach et al. 2016), which, like Pex5p, relies on the same classical import machinery consisting of docking complex, ubiquitination machinery and exportomer. In contrast to Pex5p, Pex9p has a high turnover rate, which underlines the role of Pex9p as a receptor for selective import and high metabolic adaptability. Additionally, the role of the conserved Cysteine 6 within Pex9p was demonstrated to be required for monoubiquitination and functional extraction from peroxisomal membrane, indicating that Pex9p acts as a cycling receptor as it was shown for other PTS receptors.

**Yelena Sargsyan** (Jutta Gärtner and Sven Thoms laboratory, University Medical Center Göttingen, Germany) used genetically encoded Ca^2+^ indicators (GECI) in HeLa cells, neonatal rat cardiomyocytes (CM) and human induced pluripotent stem cell (hiPSCs) derived CM to elucidate the calcium handling of peroxisomes. Several different peroxisomal Ca^2+^ sensors were developed and applied for single cell-based intraperoxisomal Ca^2+^ imaging after pharmacological stimulation (Sargsyan et al. 2020). Yelena demonstrated that in peroxisomes of HeLa cells the depletion of ER calcium stores and the Ca^2+^ influx across the plasma membrane leads to an uptake of Ca^2+^ into peroxisomes. Peroxisomal contact with ER may be a relevant player in Ca^2+^ transport to peroxisomes (Sargsyan and Thoms 2020). Further, peroxisomes in CM take up Ca^2+^ on a beat-to-beat manner. Thus, she was able to show that peroxisomal and cytosolic calcium signals are tightly interconnected, which leads to the assumption that peroxisomes play an important role in shaping cellular Ca^2+^ dynamics in CM.

**Daniëlle Swinkels** (Myriam Baes laboratory, KU Leuven, Belgium) used a mouse model lacking the D-specific multifunctional protein 2 (Mfp2-/- mice) (Baes et al. 2000) to investigate whether reduced plasma docosahexaenoic acid (DHA) underlies the retinal degeneration of patients with peroxisomal β-oxidation deficiency. Therefore, mice were fed with a diet containing 0.1% DHA during gestation and lactation. After 4 weeks, lipidome analysis revealed a normalization of DHA-containing phospholipids in the neural retina. Additionally, Daniëlle was able to show that the retinal morphology improved, regarding to the length of photoreceptor outer segments (POS), the photoreceptor cell death, the protrusions of retinal pigment epithelial cells into the POS layer and loss of the honeycomb pattern. Thus, supplementation of DHA was able to restore the morphology of the retina, but its function was not improved.

Finally, in this session, **Mounia Tahri-Joutey** (Pierre Andreoletti and Mustapha Cherkaoui-Malki laboratory, University of Bourgogne, France) talked about the dysregulation of microglial phagocytosis and lysosomal functions in peroxisomal β-oxidation deficiencies. Mounia used Acox1- (Raas et al. 2019b) or Abcd1/d2-deficient (Raas et al. 2019a) BV 2 cell lines, which were generated by CRISPR/cas9, to investigate the impact of peroxisomal β oxidation deficiency on the expression of lysosomal proteins on the phagocytosis function in microglial cells. Thereby, she showed that several lysosomal genes like cathepsins (CATHB, CATHK) and LAMP2 are post-transcriptional and post-translational overexpressed. Additionally, the phagocytosis was enhanced in BV-2 Acox1-deficient cells, while in BV-2 Abcd1/d2-deficient cells the phagocytosis was reduced. Thus, Mounia showed that a defect in peroxisomal β-oxidation influences the phagocytosis and lysosomal function in microglial cells.

## Session 8: Chair: Wolfgang Schliebs, Ruhr-University Bochum, Germany

**Laura C. Terrón-Camero** (María C. Romero-Puertas and Luisa M. Sandalio laboratory, CSIC, Granada, Spain) reported about a peculiar role of Nitric oxide (NO) in the proliferation of plant peroxisomes. This process, which sometimes begins with the formation of peroxisomal membrane extensions (peroxules), can be induced by cadmium stress. Interestingly, cadmium-induced proliferation is highly compromised in nia1 nia2 mutants, which have lower NO levels than wildtype. NO-related mutants also display alterations in the number of organelles, peroxisomal-dependent signaling and peroxisomal ROS metabolism. These phenotypes correlate with changes of the pattern of oxidative modification and S-nitrosylation of catalase, one of the main antioxidant enzymes in peroxisomes. All in all, the presented results suggest that a finely tuned balance between ROS and NO is necessary to regulate responses to cadmium stressed seedlings (Terrón-Camero et al. 2020).

**Esther Nuebel** (Jared Rutters laboratory, University of Utah, USA) proposed a novel potential therapy for patients with peroxisomal biogenesis disorders (PBD). The authors of this study explored the fate of peroxins in Zellweger cell lines and found that these proteins, which are produced at high levels, accumulate on the mitochondrial membrane, impairing respiration and ATP synthesis. It is known for a long time that dysfunction of mitochondria is also a hallmark of most severe PBDs. Remarkably, mitochondrial function in PEX3 and PEX16 deficient fibroblasts derived from human patients with PBD could be rescued by overexpressing ATAD1, an AAA–ATPase that functions in mitochondrial quality control. Although it is still unclear whether mitochondrial defects directly contribute to PBD pathophysiology or are a secondary result of nonfunctioning peroxisomes, protecting mitochondrial function by supporting quality control pathways seems to be a promising target for an alternative therapy of peroxisomal disorders (Nuebel et al. 2020).

**Takehiko Yasumitsu** (Yukio Fujiki laboratory, Kyushu University, Fukuoka, Japan) identified Serine 232 of human PEX14 as phosphorylation site in mitotically arrested cells (Yamashita et al. 2020). The increase of cytosolic catalase steady state level at the M-phase could protect DNA from highly reactive oxygen species including H_2_O_2_ upon breakdown of the nuclear envelope. While peroxisomal import of several PTS1 proteins does not seem to be affected by phosphomimetic mutations at the Serine 232, the import efficiency of catalase with a non-canonical targeting signal sequence is suppressed in CHO cells, likewise counteracting against ROS species including H_2_O_2_ (Okumoto et al. 2020).

**Francesca Di Cara** (Dalhousie University, Canada) presented latest results from a collaborative effort to analyze the relationship between peroxisome activity and macrophage functions in immunity during infection, like phagocytosis and production of inflammatory and anti-microbial molecules. Francesca and colleagues used the fruit fly *Drosophila melanogaster* and determined that peroxisomes are needed to form distinct membrane phospholipid nanodomains to activate RhoA-dependent signals that in turn drive macrophage activation in response to microbial challenge and inflammatory environments. This peroxisome–phospholipid–RhoA signaling axis is conserved in macrophage activation in mammals. These studies show that peroxisomes control distinct lipid metabolic pathways necessary for macrophage activation and define peroxisomal activity as a potential metabolic marker for immune disorders.

**Derrick Cheng** (Peter Kim laboratory, University of Toronto, Canada) discussed how a family of lipid transfer proteins called oxysterol-binding protein-related proteins (ORPs) are involved in promoting peroxisome maintenance at peroxisome–ER contact sites. The group of Peter Kim has previously discovered the proteins that maintain the peroxisome–ER contacts, which are required for lipid metabolism and peroxisome growth (Hua et al. 2017). Both these functions require unknown lipid transfer mechanisms. To this end, Derrick and colleagues identified a lipid transport protein from the ORP family that affects peroxisome growth. Using confocal microscopy, they demonstrated that this ORP localizes to sites of ER–peroxisome contact and is essential for peroxisome maintenance.

## Conclusion of the meeting

After the last session, **Einat Zalckvar** presented the Peroxi-Club (maintained by Einat Zalckvar from the Schuldiner laboratory), which provides an online platform for interested peroxisome researchers to meet and to attend exciting monthly lectures on peroxisome biology. The Peroxi-Club is very well recognized and adds to successful joint ventures like the above mentioned European Training Network ‘PerICo’ (Peroxisome Interactions and Communication) coordinated by Ida van der Klei or the German Research Unit ‘PerTrans’ (Structure and Function of the Peroxisomal Translocon) coordinated by Ralf Erdmann as hallmarks for the excellent collaboration within the peroxisome community. After Einat’s presentation, the audience separated into breakout rooms for discussion of peroxisome-specific topics. The session on ‘Metabolism of Peroxisomes’ was chaired by Ron Wanders (Amsterdam, NL) and Marc Fransen (Leuven, BE), ‘Peroxisome Biogenesis by Einat Zalckvar (Rehovot, IL) and Michael Schrader (Exeter, UK), ‘Peroxisomal Diseases’ by Hans Waterham (Amsterdam, NL) and Johannes Berger (Vienna, AT). The last talk of the meeting was the above-mentioned Young Investigator Award Lecture ‘Deubiquitinating enzyme USP30 maintains basal peroxisome abundance by regulating pexophagy’ presented by Victoria Riccio (University of Toronto, Canada).

During the meeting, the audience had the chance to vote for the best talks in an online election, which was excellently organized by Maya Schuldiner (Weizmann Institute of Science, Israel). The Awards were sponsored by the Ruhr-University of Bochum and the winners were announced at the end of the meeting. The prize for the best oral presentation was awarded to **Eden Yifrach** (Weizmann Institute of Science, Israel), the award for the second place was given to **Kyla Germain** (University of Toronto, Canada) and the award for the third place was shared by **Esther Nuebel** (University of Utah, USA) and **Pascal Lill** (Max-Planck Institute Dortmund, Germany).

Finally, possible places for the next OEPM were presented and after the voting of the audience, it was announced that the OEPM 2022 will be organized by **Jorge Azevedo** in Porto (Portugal).

Despite the fact that the 7th OEPM took place online, the meeting was very successful as it brought together peroxisome researchers from all over the world and offered the opportunity to young researchers to present their cutting edge science to the peroxisome community.
